# Analysis of the Influence of Macro-Bending Loss in Single-Mode Optical Fibers on OFDR Signal Quality

**DOI:** 10.3390/s25226983

**Published:** 2025-11-15

**Authors:** Xiaoxi Qu, Fuqiang Ma, Shiyuan Zhao, Lei Yang, Zhanjun Wu, Bingzhi Chen

**Affiliations:** 1 School of Mechanical Engineering, Dalian Jiaotong University, Dalian 116028, China; syquxiaoxi@djtu.edu.cn (X.Q.); chenbingzhi06@126.com (B.C.); 2 Dalian Institute of Measurement and Control Technology, Dalian 116013, China; ma_fu_qiang@126.com; 3 School of Optoelectronic Engineering and Instrument Science, Dalian University of Technology, Dalian 116024, China; zhaoshiyuan@dlut.edu.cn; 4 School of Mechanics and Aerospace Engineering, Dalian University of Technology, Dalian 116024, China; 5 School of Materials Science and Engineering, Dalian University of Technology, Dalian 116024, China; wuzhj@dlut.edu.cn

**Keywords:** single-mode optical fiber, macro-bending loss, optical frequency domain reflectometry (OFDR), signal-to-noise ratio (SNR)

## Abstract

This study investigates the influence of optical loss induced by the macro-bending of optical fibers on the signal quality of an optical frequency-domain reflectometry (OFDR) system. First, the finite element software COMSOL 5.3 was used to perform numerical simulations of the optical loss of single-mode fibers under different bending radii. The simulations revealed that when the bending radius is relatively small, the optical loss exhibits oscillation as the bending radius varies. Next, an optical backscatter reflectometer (OBR) was employed to measure the optical loss of the optical fiber under different bending radii and numbers of bending loops. The experimental results showed good consistency with the simulation results, and the variation law of optical loss under different bending radii and numbers of bending loops was clarified. An OFDR strain demodulator was used to demodulate the strain signals under loaded conditions with different fiber bending radii and numbers of bending loops. It was found that when the cumulative optical loss increases to a certain threshold, the demodulated signal quality degrades significantly—this confirms that macro-bending loss directly impacts the SNR of OFDR output signals. The findings of this study provide practical guidance for the bending-oriented deployment of optical fiber sensors, which was successfully validated through a real-world structural strain monitoring case.

## 1. Introduction

Optical fiber sensors exhibit numerous advantageous characteristics, including small volume, low weight, strong integrability, and adaptability to extreme environments. They are widely employed in structural monitoring across fields such as medicine, aerospace, civil engineering, and automotive engineering [1,2,3,4,5,6,7,8,9]. An optical fiber primarily consists of three components: the core, the cladding, and the outer coating [10]. When an optical fiber is situated in an external strain field or temperature field, variations in strain or temperature induce changes in the fiber’s internal refractive index, which in turn alter the corresponding Rayleigh scattering signal. By measuring the optical frequency of this signal, the detection of strain or temperature variations can be realized.

However, in the health monitoring of large-scale structural equipment and optical communication systems, it is difficult to ensure that optical fibers are deployed along a fully straight path. Their deployment paths inevitably contain multiple bends. When light waves propagate in an optical fiber, the intensity of optical power gradually decreases as the transmission distance increases. The fiber causes attenuation of the light waves, which is referred to as optical loss or optical attenuation. The reason for this loss is that when the optical fiber is bent, part of the power that originally propagates in the form of guided modes in the fiber core is converted into the power of radiation modes and overflows from the fiber core, resulting in loss [11,12]. The emergence of optical loss degrades the output Rayleigh scattering signal and reduces the accuracy of monitoring results [13,14]. Therefore, full consideration should be given to minimizing such losses during optical fiber deployment [15].

The bending behavior of optical fibers is categorized into two types: macro-bending and micro-bending. Macro-bending typically refers to a scenario where the fiber’s axis forms a circular curve, with a bending diameter far larger than the fiber’s own diameter. At the circular bend, light radiates outward from the fiber’s interior, penetrates the cladding and outer coating, and leaks into the surrounding air—this process results in macro-bending loss [16]. Micro-bending, by contrast, is usually caused by random perturbations of the fiber core along the fiber’s axis, and its bending diameter is smaller than the fiber’s own diameter. The spacing between adjacent micro-bends is typically several micrometers; along the fiber’s length, light radiates outward from the fiber’s interior intermittently, leading to micro-bending loss [17].

To reduce optical fiber bending loss, researchers have conducted in-depth research on various factors affecting it and continue to explore effective solutions. For macro-bending, key factors influencing optical loss include optical wavelength, fiber bending radius, and number of bending loops [18,19,20]. In related research, Zheng et al. [21] derived a formula for calculating the bending loss of individual high-order modes via the perturbation method. Cherpak et al. [22] computed the optical loss coefficient of metal-coated fibers across a wide wavelength range by measuring changes in the coating’s thermistor response induced by laser radiation propagating through the fiber. Bulatov et al. [23] investigated the relationship between optical loss in silica multimode fibers and two critical fiber parameters: outer diameter and cladding thickness. Pieter et al. [24] explored how the fiber cladding’s material properties and boundary conditions affect optical loss using numerical simulations. Meng et al. [25] developed a macro-bending loss modulation method and explored the relationship between the shape of the helical structure and the macro-bending loss of optical fibers. For micro-bending, Jin et al. [26] established an analytical model for micro-bending behavior in optical fibers with arbitrary refractive indices—with a specific focus on characterizing micro-bending loss. Gu et al. [27] proposed a noise-limited sensitivity analysis model for fiber optical frequency-domain reflectometry (OFDR), utilizing the sensitivity and signal-to-noise ratio (SNR) variations across different positions in the optical fiber to quantify the sensing accuracy of the OFDR system.

In summary, the occurrence of optical loss impairs the output Rayleigh scattering signal and thereby reduces the accuracy of monitoring results. However, a research gap remains regarding the mechanism by which optical loss impacts the quality of Rayleigh scattering signals—specifically, how loss quantitatively and mechanistically degrades signal integrity. To address this issue, the present study investigates the influence of macro-bending loss on the signal quality of single-mode OFDR using a combined approach of numerical simulations and experiments. First, numerical simulations were performed to calculate the macro-bending loss of optical fibers under varying bending radii, establishing a quantitative relationship between bending geometry and loss magnitude. Subsequently, experimental tests were conducted to achieve three key objectives: (1) further exploring the influence of the number of bending loops on optical loss, (2) quantifying the impact of different optical loss levels on the quality of OFDR output signals, and (3) utilizing the macro-bending loss research findings to guide sensor path deployment in a real-world application scenario. Given that users of commercial OFDR systems typically cannot modify the built-in demodulation algorithms, this study provides pragmatic value by offering informed guidance for the optimal placement and application of optical fibers in macro-bend-prone environments.

## 2. Theory of Macro-Bending Loss in Optical Fibers

### 2.1. Principle of Strain Measurement Based on OFDR

**Figure 1:**
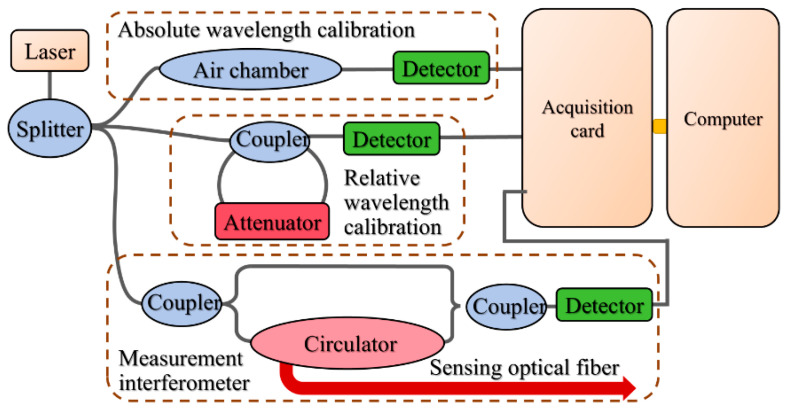
Basic structure of the OFDR system.

The optical frequency-domain reflectometry (OFDR) system employs a linearly frequency-swept laser as its light source to enable coherent detection. This design maps two key pieces of information: the backscattering position within the optical fiber to the frequency of the beat frequency signal, and the backscattering amplitude to the signal’s power spectral density. The basic structure and working principle of the OFDR system are illustrated in Figure 1. The light source emits a detection laser with a frequency that varies linearly over time. This laser is split into two separate paths by a beam splitter: one path directs the light wave into a measurement fiber and a reflector; the other path injects light into the fiber under test, generating a back Rayleigh scattering signal. Subsequently, the two optical signals are combined and detected by a photodetector, which outputs a beat frequency signal. Applying a Fourier transform to this beat frequency signal enables the mapping of Rayleigh scattering positions along the fiber to specific beat frequencies.

**Figure 2:**
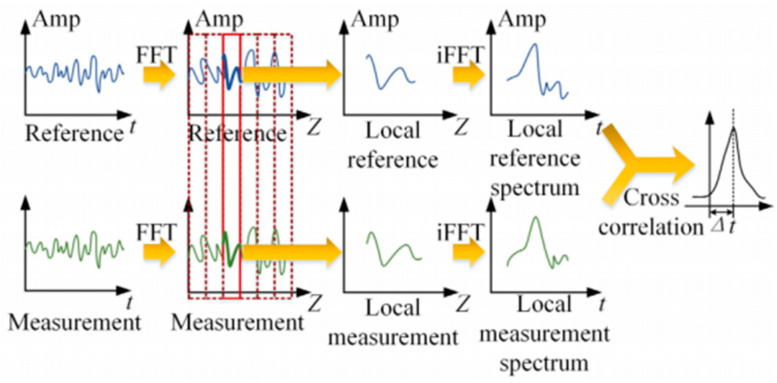
Coherent demodulation steps of Rayleigh scattering spectrum.

Traditional OFDR technology is mainly applied to short-distance optical link diagnosis and high-spatial-resolution measurement of optical devices. The distributed parameter measurement technology based on the OFDR principle is developed on the basis of classical OFDR, incorporating an additional signal processing procedure for distributed parameter calculation and a coherent demodulation method specific to Rayleigh scattering spectra. The steps of coherent demodulation for Rayleigh scattering spectra are shown in Figure 2. First, the OFDR system is used to collect the Rayleigh scattering spectra of the optical fiber in its initial state and deformed state. These two sets of spectra serve as the reference Rayleigh scattering spectrum signal and the measurement Rayleigh scattering spectrum time-domain signal, respectively, with the ordinate representing the amplitude of the Rayleigh scattering spectrum. Next, Fast Fourier Transform (FFT) is applied to convert the wavelength-domain signals into signals in the fiber distance domain, yielding the reference Rayleigh scattering spectrum and measurement Rayleigh scattering spectrum in the distance domain. The fiber under test is divided into several sensing units, namely measurement subsets. A subset window of length *m* is then used to partition both the reference and measurement Rayleigh scattering spectra (in the distance domain) into *n* subsets. Finally, the cross-correlation function is employed to compare the similarity between the reference subset (from the reference Rayleigh scattering spectrum) and the target subset (from the measurement Rayleigh scattering spectrum). This comparison yields the offset of the Rayleigh scattering spectrum, which is further converted into strain or temperature information. After the optical signal entering the optical fiber undergoes splicing loss, Fresnel reflection, and bending loss, the energy of its Rayleigh scattering spectrum signal will decrease to a certain extent [17].

### 2.2. Calculation Formula of Macro-Bending Loss

The currently widely used calculation formula for macro-bending loss of optical fibers was first proposed by Marcuse in 1976. This model treats the optical fiber as a core-infinite cladding model and derives the calculation formula for the macro-bending loss factor as follows [28]:

(1) $$2\alpha =\frac{\sqrt{\pi }\kappa^{2}\exp[-\frac{2}{3}(\gamma^{3}/\beta_{g}^{2})R]}{e_{\nu }\gamma^{3/2}V^{2}\sqrt{R}K_{\nu -1}(\gamma a)K_{\nu +1}(\gamma a)}$$

(2) $$k=\frac{2\pi }{\lambda }$$

(3) $$\gamma =\sqrt{\beta_{g}^{2}-{n_{2}}^{2}k^{2}}$$

(4) $$V^{2}=k^{2}\alpha^{2}({n_{1}}^{2}-{n_{2}}^{2})$$

where *α* is defined as the amplitude loss coefficient of the guided wave, and 2*α* is the optical loss coefficient, *λ* is the detection light wavelength, *n*_1_ and *n*_2_ are the refractive indices of the fiber core and cladding, respectively, *k* is the free-space propagation constant, *β_g_* is the propagation constant of the guided mode in the straight guide, *R* is the curvature radius.

In practical applications, optical fibers not only consist of a core and a cladding but also include one or two coating layers that provide mechanical protection. Therefore, this theoretical model can only be used to predict scenarios where the coating layers are removed and an absorption layer is added. The structure of “core-cladding-absorption layer” can be approximately regarded as a core-infinite cladding model. It can be seen from Equations (1)–(4) that the macro-bending loss of an optical fiber is related to the bending radius, wavelength, core radius, core refractive index, and cladding refractive index.

## 3. Numerical Simulation of Macro-Bending Loss

### 3.1. Finite Element Method

In this study, the commercial software COMSOL Multiphysics was employed to solve for the effective mode refractive index of a bent optical fiber, and the imaginary part of the refractive index is used to calculate the macro-bending loss.

For a straight optical fiber, first-order mode analysis is conducted on the fiber cross-section. The electromagnetic wave propagates along the z-direction, with its expression given as follows:

(5) $$E\left(x,y,z,t\right)=E\left(x,y\right)e^{j\left(\omega t-\beta z\right)}$$

where *ω* is the angular frequency, and *β* is the propagation constant.

The eigenvalue equation of the electric field *E* is solved by the following Helmholtz equation:

(6) $$\nabla \times \left(\nabla \times E\right)-k_{0}^{2}n^{2}E=0$$

By solving the above equation, the eigenvalue λ=β can be obtained, and the effective refractive index is $n_{eff}=\lambda /k_{0}$.

For a bent optical fiber, second-order mode analysis is conducted on the two-dimensional axisymmetric geometry of the fiber cross-section. In this scenario, the wave propagates along the *φ* direction, and the electric field is expressed as follows:

(7) $$E\left(r,\phi ,z,t\right)=E\left(x,y\right)e^{j\left(\omega t-\beta r_{0}\phi \right)}$$

where $r_{0}$ denotes the curvature radius of the bent fiber.

For this scenario, the Helmholtz equation is solved to derive the eigenvalue $\lambda =\beta r_{0}$. Consequently, both the reference refractive index input into the eigenvalue solver and the effective refractive index obtained from the solver are scaled based on the radius $r_{0}$, specifically: $n_{eff}=\lambda /r_{0}/k_{0}$.

**Figure 3:**
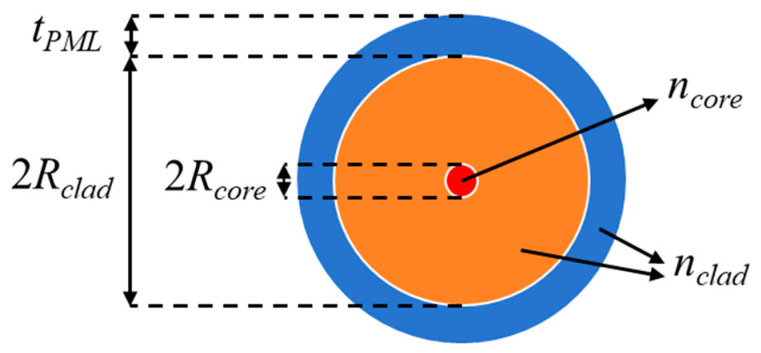
Finite element model of the optical fiber.

The simulation model employs a core-cladding infinitely extended structure. The bent optical fiber exhibits central axial symmetry, and the entire fiber can be generated by rotating a specific cross-section. To investigate the influence of bending radius on optical loss, the bending radius is treated as a variable parameter via parametric modeling. A scattering boundary condition is applied to the outer boundary of the fiber cladding to absorb the optical field that is vertically incident on the boundaries of the cladding. A perfectly matched layer (PML) is adopted to mimic an “infinite cladding”. To enhance the accuracy of the simulation results, an ultra-fine mesh size is employed. The finite element model is illustrated in Figure 3.

The optical fiber used in this study is polyimide-coated single-mode fiber, and its geometry and material properties are detailed in Table 1.

### 3.2. Results Analysis

**Figure 4:**
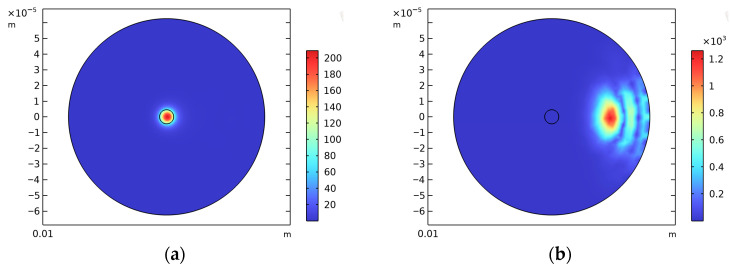
Electric field distribution diagrams: (**a**) Fundamental mode. (**b**) Pseudo mode.

Figure 4 shows the simulated electric field distribution of the optical fiber. Different modes correspond to different effective refractive index results: as presented in Figure 4a, the electric field is confined within the fiber core, which corresponds to the fundamental mode; in Figure 4b, the electric field is concentrated in the cladding, which is identified as a pseudo mode. Using the imaginary part of the effective refractive index under the fundamental mode, the optical loss per bend loop (in dB/loop) at the given bending radius can be calculated via Equation (8) [29]:

(8) $$2\alpha =\frac{20}{In(10)}\frac{2\pi }{\lambda }Im\{n_{eff}\}\pi \cdot 2R_{b}$$

**Figure 5:**
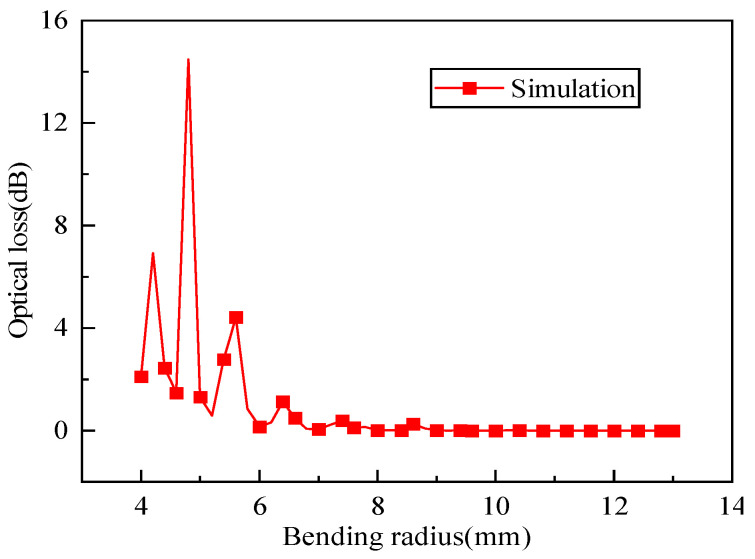
Relationship between optical loss and bending radius by simulation.

The calculated optical loss curve of the optical fiber per unit length caused by different bending radii is shown in Figure 5. As observed, when the optical fiber’s bending radius is small, the optical loss is large; overall, the optical loss exhibits a decreasing trend as the bending radius increases. When the bending radius exceeds 7 mm, the loss approaches zero. Additionally, when the bending radius is relatively small, there is an oscillation of the optical loss value. This oscillation of optical loss with the variation in bending radius is caused by the coupling between the Whispering-gallery modes propagating in the cladding and coating layers and the fundamental mode in the core. The period of the oscillation is determined by the bending shape, as well as the optical and physical properties of the fiber’s cladding and coating layers [20].

## 4. Experimental Verification

### 4.1. Optical Loss Test

**Figure 6:**
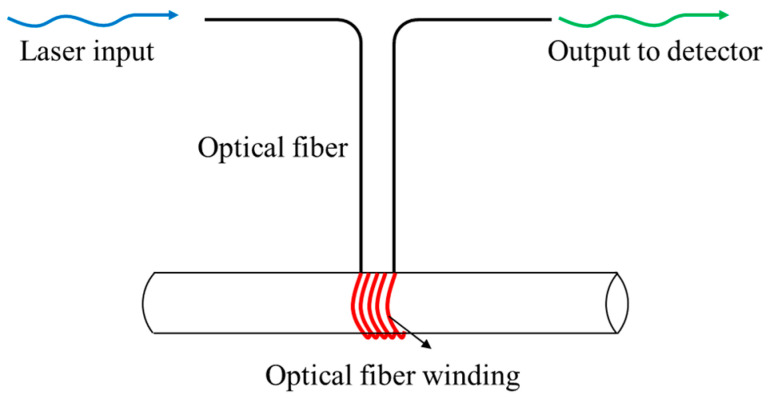
Schematic diagram of winding equipment for measuring optical loss.

To investigate the influence of fiber bending radius on optical loss, an optical backscatter reflectometer (OBR) developed by LUNA (USA) based on OFDR technology was used for experimental measurement of optical loss. The optical fiber used in the experiment is a polyimide-coated standard SMF-28 single-mode fiber manufactured by Fiberlogix Intl. Ltd.(Watford, United Kingdom). As shown in Figure 6, the optical fiber was wrapped around cylindrical tubes of different diameters, so that the bending radius of the optical fiber starts at 4 mm and increases in increments of 0.5 mm.

**Figure 7:**
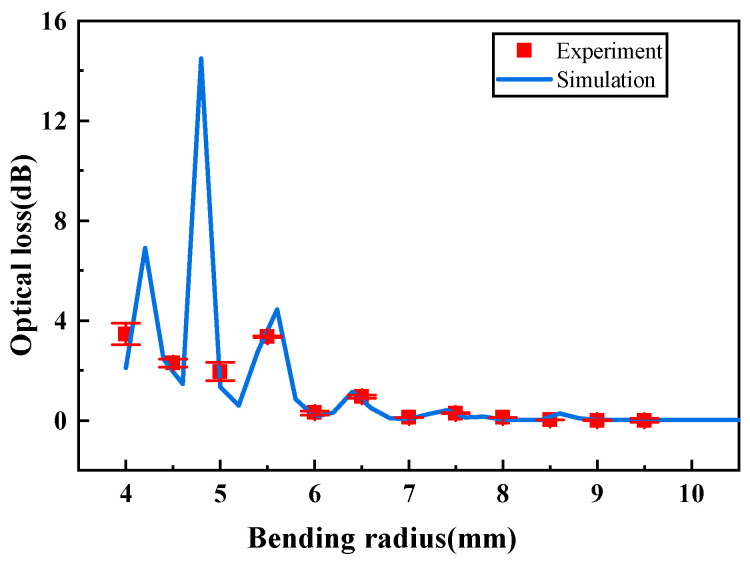
Comparison between experimental and simulation results of optical loss.

The optical loss per bending loop under different bending radii was measured experimentally, with the results shown in Figure 7. It can be seen that the experimental results are in good agreement with the simulation results, and the variation trend of optical loss with the fiber bending radius is consistent, thus verifying the reliability of the finite element simulation model.

**Figure 8:**
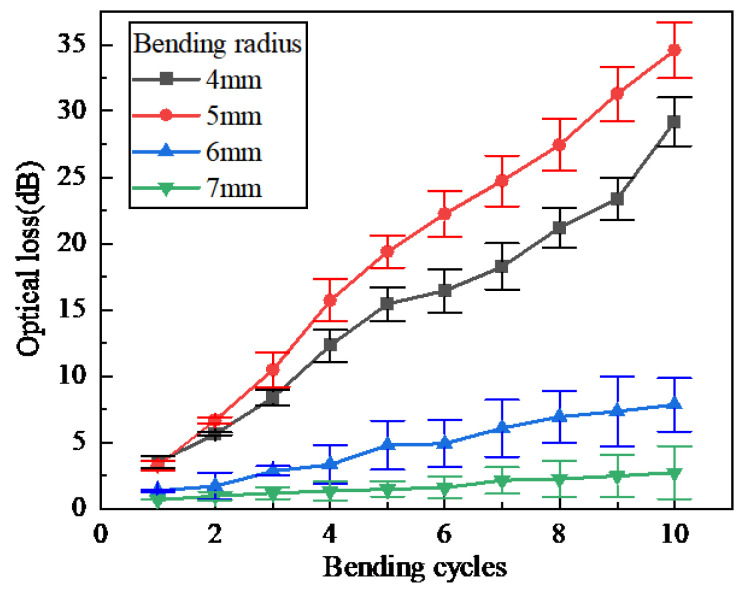
Optical loss under different numbers of bending loops.

The same method was used to further investigate the influence of the number of bending loops on optical loss. The optical fiber was wound into different numbers of loops, and optical loss was measured under a fixed bending radius and varying numbers of bending loops. From the obtained results in Figure 8, it is found that the optical loss value generally exhibits an increase trend as the number of bending loops increases. Specifically, when the fiber bending radius is small, the optical loss increases more significantly with the increase in the number of bending loops; when the fiber bending radius is large, the optical loss increases at a slower rate as the number of bending loops increases. Additionally, when the bending radius exceeds 7 mm, the optical loss value is close to zero.

It is worth noting that a smaller bending radius does not necessarily correspond to a larger optical loss value. As can be seen from Figure 8, when the number of bending loops ranges from 2 to 10, the optical loss under a bending radius of 4 mm is smaller than that under a bending radius of 5 mm. This further confirms the oscillation phenomenon of optical loss observed in Figure 5.

### 4.2. Influence of Optical Loss on Signal Quality

**Figure 9:**
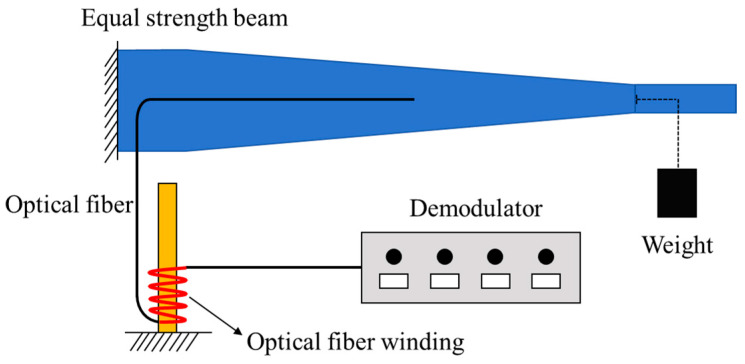
Schematic diagram of equal strength beam loading experiment.

To investigate the influence of macro-bending loss on OFDR signal demodulation, a static loading experiment using an equal-strength beam was conducted. As shown in Figure 9, the optical fiber was bonded to the upper surface of the equal-strength beam. Load was applied to the equal-strength beam by using weight, and the optical fiber was wrapped around a cylindrical tube for a specified number of loops before being connected to the demodulator. By setting different cylindrical tube diameters and numbers of winding loops, the optical loss values were measured to further study the influence of optical loss on signal quality.

**Figure 10:**
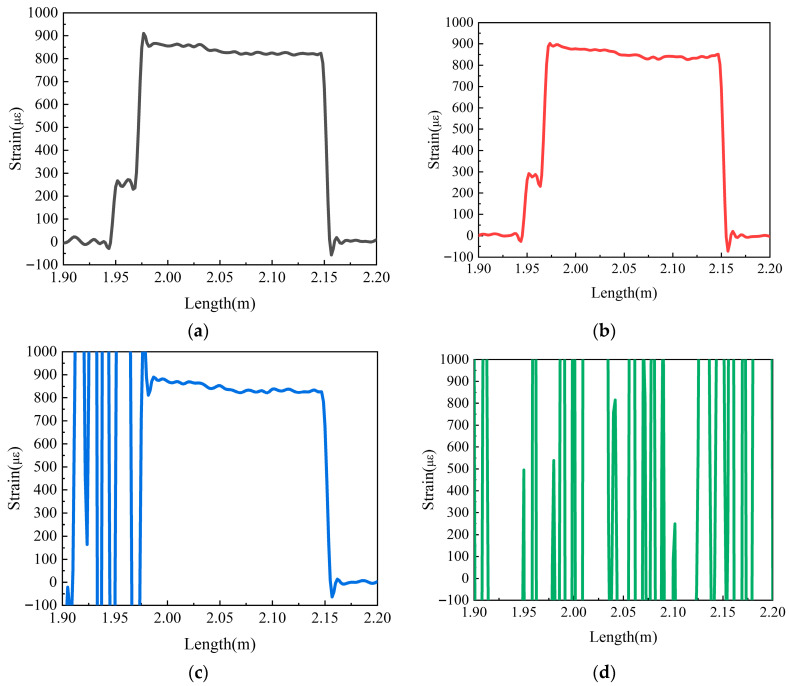
Strain response under different numbers of bending loops (bending radius = 4 mm): (**a**) 1 loop; (**b**) 2 loops; (**c**) 5 loops; (**d**) 10 loops.

Figure 10 presents the strain measurement results under the conditions of a constant load (2 N), a fixed bending radius of 4 mm, and varying numbers of bending loops. It can be observed that when the optical fiber is bent for 1 or 2 loops, the strain signal is relatively good; when the number of bending loops increases to 5, the strain signal begins to deteriorate, manifesting as a decrease in the SNR; and when the number of bending loops reaches 10, strain demodulation can no longer be achieved. This indicates that the optical loss caused by macro-bending has a significant impact on the signal quality of the optical fiber.

**Figure 11:**
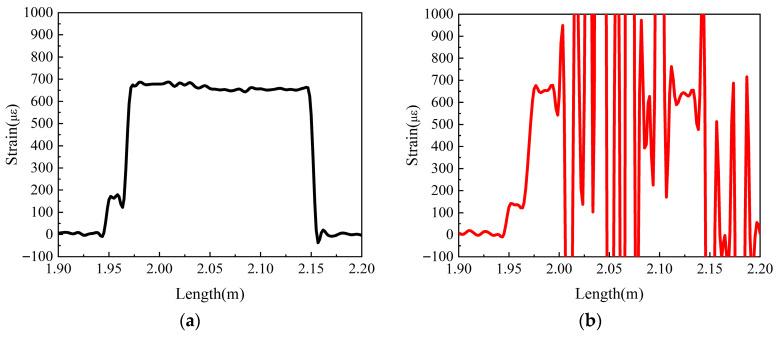
Strain response under different numbers of bending loops (bending radius = 5 mm): (**a**) 1 loop; (**b**) 2 loops.

Figure 11 shows the strain measurement results under the conditions of a constant load (2 N), a fixed bending radius of 5 mm, and varying numbers of bending loops. It can be observed that when the number of bending loops is 1, the strain measurement of the optical fiber is not affected; however, when the number of bending loops increases to 2, the strain demodulation results have already become very poor. Compared with Figure 10, this result further indicates that the optical loss of the fiber at a bending radius of 5 mm is greater than that at a bending radius of 4 mm.

**Figure 12:**
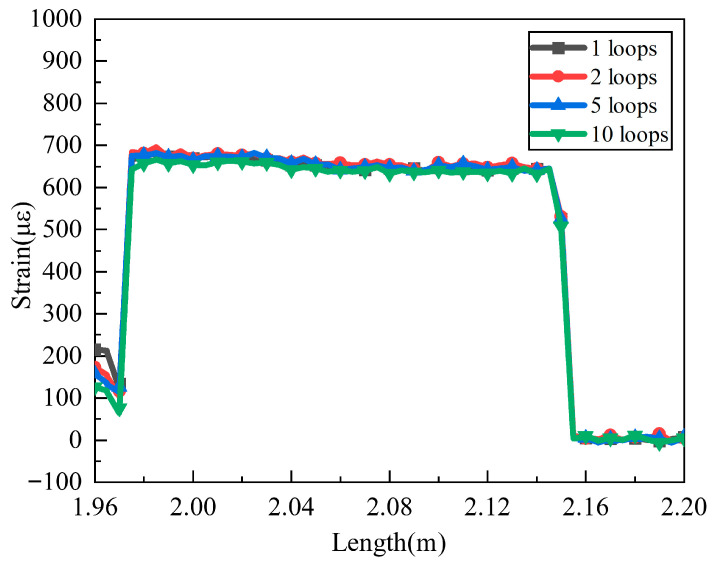
Strain response under different numbers of bending loops (bending radius = 6 mm).

Figure 12 illustrates the strain measurement results under the conditions of a constant load (2 N), a fixed bending radius of 6 mm, and varying numbers of bending loops. For the OBR series produced by Luna, the system background noise is expressed in terms of sensitivity, which is approximately −118 dB/mm. In contrast, the Rayleigh scattering signal intensity of standard SMF-28 optical fiber is −100 dB/mm. Given this, the bidirectional limit for optical fiber loss must not exceed 9 dB [30]. As can be seen from Figure 8, when the bending radius is 6 mm, the optical loss corresponding to 10 bending loops is still less than 9 dB; therefore, the influence of bending loss on signal quality remains at a low level. This low-loss condition leads to a high SNR of the strain signal.

### 4.3. Application

**Figure 13:**
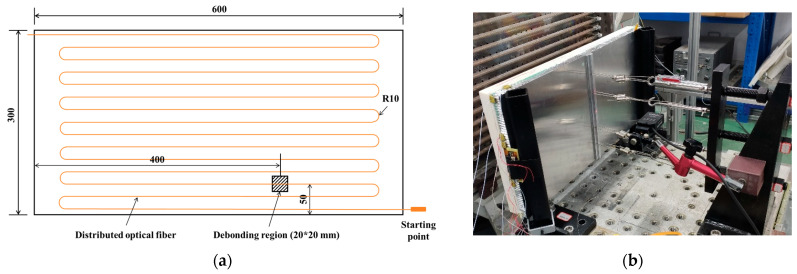
Damage monitoring of a honeycomb sandwich panel: (**a**) Schematic diagram of the structure and sensor deployment. (**b**) Three-point bending test.

To verify the guiding role of the above research results for the bending deployment of optical fiber sensors, an experiment was conducted on a 600 mm × 300 mm aluminum honeycomb sandwich panel. As illustrated in Figure 13a, a 20 mm × 20 mm debonding zone was artificially introduced between the panel and the core. A distributed optical fiber sensor network was embedded during fabrication to monitor strain signals from the structure. In order to accurately locate the prefabricated damage area, a multi-bend optical fiber layout was designed. Guided by the findings of this study—which indicate that bending loss becomes negligible when the bending radius exceeds 7 mm—a conservative bending radius of 10 mm was adopted. According to the simulation results in Section 3.2, the bending loss for a single loop at this radius is 0.0216 dB. The designed path contains 14 semicircular bends (equivalent to 7 full loops), resulting in a total bending loss of only 0.1512 dB—well below the system threshold of 9 dB. A three-point bending test was carried out under displacement-controlled loading, as shown in Figure 13b.

**Figure 14:**
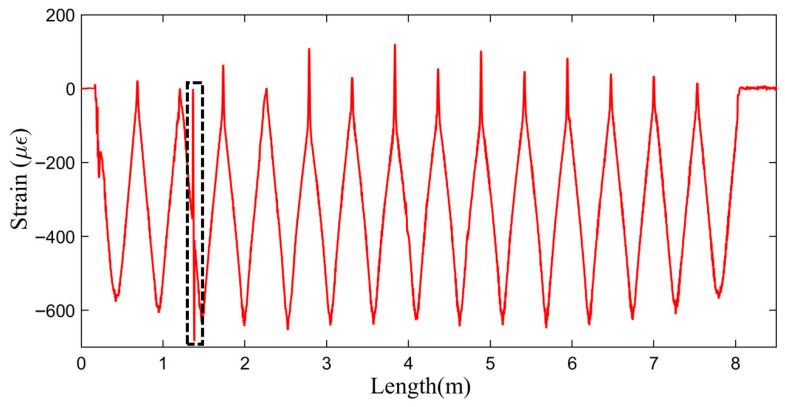
Strain data of the distributed optical fiber.

Under three-point bending, the distributed optical fiber provided continuous strain measurements at the honeycomb/panel interface, as depicted in Figure 14. The demodulated signals show high clarity, free from significant noise and with a high SNR. A pronounced strain transition is evident in the fiber segments crossing the debonding region (as indicated within the black dashed box). This result confirms the feasibility of the multi-bend sensor deployment strategy and its reliability in delivering high-SNR strain monitoring.

## 5. Conclusions

This study employed numerical simulation and experimental methods to investigate the relationship between bending radius, number of bending loops and the macro-bending loss of single-mode optical fibers, as well as the impact of macro-bending loss on OFDR signal quality. The key conclusions are as follows:

(1) COMSOL-based simulations of optical loss revealed that the macro-bending loss per loop of a single-mode optical fiber does not decrease linearly with increasing bending radius; instead, the loss exhibits oscillatory behavior under small bending radius, caused by the coupling between the Whispering-gallery modes propagating in the cladding and coating layers and the fundamental mode in the core. As the bending radius increases to a critical value (~7 mm), the optical loss approaches zero.

(2) Optical loss testing experiments demonstrated that, under a fixed bending radius, the total macro-bending loss increases linearly with the number of bending loops. When the bending radius exceeds a critical threshold (~7 mm), the optical loss tends toward zero—indicating that large enough bending radii effectively mitigate macro-bending-induced loss. The experimentally measured data are in good agreement with the simulated optical loss results, validating the simulation model’s reliability.

(3) Analysis of OFDR signal quality showed that when the cumulative macro-bending loss is less than 9 dB, it has a small impact on the signal quality of OFDR, resulting in a high SNR. In contrast, when the cumulative loss increases to a certain level (>9 dB), the output signal quality degrades significantly, accompanied by a notable decrease in SNR. This confirms that macro-bending loss directly affects the SNR of OFDR output signals. A multi-bend optical fiber sensor layout was designed and experimentally validated, achieving structural strain monitoring with a high SNR.

In summary, the simulation and experimental methods proposed in this study provide an effective approach to characterize the macro-bending loss of single-mode optical fibers and evaluate OFDR signal demodulation quality. The findings offer practical guidance for the bending-based deployment of optical fiber sensors, helping to optimize sensor layout and ensure reliable performance in scenarios involving optical fiber bending.

## Figures and Tables

**Table 1:** Geometry and material parameters of the optical fiber.

*R*_core/_μm	*R*_clad/_μm	*t*_PML/_μm	*n*_core_	*n*_clad_	*λ*/μm
4.15	62.5	15.5	1.4507	1.4440	1.60

## Data Availability

The raw data supporting the conclusions of this article will be made available by the authors on request.
